# Social Media Influence on Patient Expectations in Esthetic and Restorative Dental Treatment: A Hospital-Based Cross-Sectional Study

**DOI:** 10.7759/cureus.117372

**Published:** 2026-10-02

**Authors:** Purvansh Manocha, Divia A Rajan, Christalin R., Subija K Narayanan Kutty, Jinu George, Dhanya Shaji

**Affiliations:** 1 Conservative Dentistry and Endodontics, Government Dental College, Thrissur, Thrissur, IND; 2 Endodontics, Govt Dental College, Thrissur, Thrissur, IND; 3 Conservative Dentistry, Government Dental College, Thrissur, Thrissur, IND

**Keywords:** esthetic dentistry, patient expectation, restorative dentistry, social media, treatment decision-making

## Abstract

Objective

This cross-sectional study aimed to evaluate the influence of social media on patient expectations regarding esthetic and restorative dental treatment among patients attending the Outpatient Department of Conservative Dentistry.

Methodology

A hospital-based cross-sectional observational study was conducted among 280 participants aged 18-40 years attending the Outpatient Department of Conservative Dentistry. Participants were selected using sequential sampling. Data were collected using a validated, self-administered questionnaire assessing sociodemographic characteristics, social media usage patterns, and perceptions toward dental treatments. Descriptive statistics were used to summarize the data, and associations between responses and study variables were analysed using the Chi-squared test.

Results

Social media served as a platform for patient awareness and information dissemination; however, its influence on actual treatment decisions appeared to be moderate. Exposure to dental treatment-related content on social media was common, with Instagram being the most frequently used platform among the younger age group.

Conclusion

Evidence-based communication by dentists can help align patient expectations with realistic clinical outcomes.

## Introduction

Currently, social media is a powerful influence that is shaping trends, impacting our thoughts, and driving consumer behavior at both the individual and societal levels. It influences our lives so much that it affects what we wear, what we eat, and even how we look at our health. Dentistry is not an exception to this. Patients nowadays do a lot of online research about the treatment options before they even step into a dental clinic. Everyday social media feeds are filled with perfect smiles, pain-free procedures, and quick-fix videos with happy patients. For someone who feels anxious about a dental treatment, that kind of content can feel comforting, but it can set unreal expectations too. Such unrealistic expectations can lead to disappointment in at least some patients. Many patients build their hopes around what they see online, only to feel disheartened when the actual procedure is more complex [1].

Social media, especially short videos, can help in educating patients about the treatment and make them less anxious [2]. Getting influenced by such content, many patients compare their own smile to what they see on social media, which leads to disappointment and they become less confident about their natural smiles. Many even opt for unnecessary cosmetic procedures like veneers or teeth whitening just to match what they had seen [3]. But on the flip side, some people may be more skeptical or cautious about the various treatment modalities advertised on social media. Visuals like videos and pictures are more powerful tools for communication, but without proper context, they can mislead people. A gap between real and digital dentistry can cause a lot of confusion [4]. This study was done to identify this gap between real and digital dentistry and detect ways to bridge the same for better patient acceptance and care.

## Materials and methods

Study design and setting

A hospital-based, cross-sectional observational study was conducted using a structured, self-administered questionnaire to assess the influence of social media on patients’ expectations regarding esthetic restorative dental treatment. The study was conducted in the Outpatient Department of Conservative Dentistry, Government Dental College, Thrissur, Kerala, India. The study setting comprised patients attending the department for routine dental consultation and treatment.

Study duration

The study was conducted over a period of 6 months. During the study period, eligible patients attending the outpatient department were screened according to the predefined inclusion and exclusion criteria and recruited sequentially until the required sample size was achieved.

Study population and participant selection

The study population consisted of patients aged 18-40 years attending the Outpatient Department of Conservative Dentistry during the study period. Patients were screened for eligibility before enrolment. Those fulfilling the eligibility criteria were informed about the purpose and procedures of the study, and written informed consent was obtained before administration of the questionnaire.

Inclusion criteria included patients attending the Outpatient Department of Conservative Dentistry, Government Dental College, Thrissur, aged between 18 and 40 years, and who were willing to participate and provide informed consent. Active users of one or more social media platforms and patients who were able to understand either Malayalam or English sufficiently to complete the questionnaire were included. Exclusion criteria included non-users of social media platforms, patients with cognitive impairment that could affect their ability to understand the questionnaire or make decisions regarding participation, patients attending the department for emergency dental care, and patients unwilling to participate in the study or unwilling to provide informed consent. A sequential sampling technique was employed. Patients attending the outpatient department during the study period were assessed consecutively for eligibility. All patients who fulfilled the inclusion criteria and did not meet any exclusion criterion were invited to participate. Recruitment was continued sequentially until the predetermined sample size was achieved.

Sample size calculation

To calculate sample size for the present study, the formula used is $n = \frac{z^2 \, p(1-p)}{d^2}$, where, p=0.86 (proportion of respondents who were influenced by dental practice social media), d=5% of p=0.043, Z=1.96 for α =0.05. n= 0.4625/0.0018=257. So, 280 people were selected for the study. Using these parameters, the calculated minimum sample size was approximately 257 participants. A total of 280 participants were ultimately included in the study to ensure that the required sample size was adequately met.

Study variables and potential confounders

The primary variables assessed included participants’ sociodemographic characteristics, social media usage patterns, and expectations regarding dental treatment. Sociodemographic variables included age, sex, educational status, occupation, and socioeconomic status. Social media-related variables included the social media platform used and the amount of time spent on social media. Potential confounding factors considered in the analysis included age, sex, educational status, occupation, socioeconomic status, type of social media platform used, and duration/time spent on social media. These variables were considered because they may influence both the extent of exposure to dental-related social media content and patients’ expectations or treatment-related perceptions. Associations between these variables and questionnaire responses were therefore assessed statistically.

Questionnaire development and data collection

Data were collected using a structured, self-administered questionnaire. The questionnaire was developed following a review of the relevant literature and existing questionnaires addressing social media use, dental treatment expectations, and the influence of social media on dental decision-making. Relevant questions from existing questionnaires were reviewed for their relevance to the objectives of the present study and were adapted to suit the study population and setting. Additional questions were incorporated to assess specific domains relevant to the present study, including social-media exposure to dental-related content, treatment expectations, treatment preferences, perceptions of esthetic and restorative procedures, trust in online information versus dental professionals, and the perceived influence of social media on treatment-related decisions. The resulting questionnaire was reviewed and validated by subject experts for content relevance and clarity. The questionnaire consisted of two sections. Section A collected sociodemographic characteristics and information regarding social media use, including the social media platforms used and time spent on social media. Section B comprised questions assessing participants’ expectations regarding dental treatment and the influence of social media on their perceptions and treatment-related expectations. Printouts of the questionnaire were given to patients fulfilling inclusion and exclusion criteria, and they were asked to give written consent to participate in the study. The patient was instructed to tick the box corresponding to their response. The self-administered format was used to minimize interviewer influence and allow participants to provide responses independently. Participants completed the questionnaire independently after receiving appropriate instructions.

Data management and statistical analysis

The completed questionnaires were checked for completeness, and the data were entered into Microsoft Excel (Microsoft Corporation, Redmond, USA) and subsequently analyzed using the Statistical Package for the Social Sciences (SPSS) (IBM Corp., Armonk, USA). Categorical variables were summarized using frequencies and percentages. Descriptive statistics were used to describe the sociodemographic characteristics, social media usage patterns, and responses to individual questionnaire items. Associations between questionnaire responses and participant characteristics, including age, sex, educational status, occupation, socioeconomic status, social media platform used, and time spent on social media, were assessed using the Chi-square test for categorical variables. Where applicable, assumptions for the Chi-square test were assessed before interpretation. A p-value <0.05 was considered statistically significant. Potential confounding variables were identified based on their relevance to social media exposure and treatment expectations and were considered during interpretation of the observed associations. Since this was a cross-sectional study, associations were interpreted as relationships between variables rather than evidence of temporal or causal relationships.

Ethical considerations and institutional ethics committee approval

The study was conducted in accordance with the ethical principles applicable to research involving human participants. Ethical approval was obtained from the Institutional Ethics Committee (IEC), Government Dental College, Thrissur, Kerala, India, before commencement of participant recruitment. The ethical approval number was IEC/M2/P2/2025/GDCTSR. Written informed consent was obtained from every participant prior to participation. 

## Results

The present observational cross-sectional study was conducted to assess the influence of social media on patient expectations regarding esthetic restorative dental treatment. The sociodemographic characteristics of the study participants, patterns of social media usage, exposure to dental-related content, perceptions regarding restorative and esthetic dental treatments, and the extent to which social media influences treatment expectations and decision-making among the participants were assessed based on the responses (Table 1 and Table 2).

**Table 1:** Demographic factors

Variable	Category	Frequency	Percentage (%)
Gender	Male	98	35.0
	Female	182	65.0
Place of Residence	Rural	56	20.0
	Urban	224	80.0
Educational Status	Illiterate	1	0.4
	Primary education	35	12.5
	Secondary education	138	49.3
	Graduate/Professional	88	31.4
	Postgraduate	18	6.4
Occupation	Professional	35	12.5
	Office worker	23	8.2
	Skilled worker	4	1.4
	Unskilled worker	10	3.6
	Housewife	47	16.8
	Student	147	52.5
	Unemployed	14	5.0

**Table 2:** Questionnaire and patient responses

Variable	Category	Frequency	Percentage (%)
How often do you see dental content on social media?	Daily	40	14.3
	Weekly	58	20.7
	Rarely	139	49.6
	Never	40	14.3
	Others	3	1.1
Which social media app do you use the most?	Instagram	118	42.1
	X (Twitter)	28	10.0
	Youtube	54	19.3
	Whatsapp	53	18.9
	Snapchat	6	2.1
	Facebook	21	7.5
Do you search on social media for your dental treatment?	Yes	96	34.3
	No	163	58.2
	Others	21	7.5
Do you follow any dentists or influencers who post about dental treatments?	Yes	77	27.5
	No	177	63.2
	Others	26	9.3
How much does social media influence your interest in restorative and aesthetic dental treatments?	Not at all	94	33.6
	Slightly	110	39.3
	Moderately	63	22.5
	Strongly	13	4.6
Which all products have you tried after seeing them on social media?	Whitening strips/Paste	36	12.9
	Charcoal toothpaste	22	7.9
	Tooth Polishers	14	5.0
	Veneers	8	2.9
	No products tried	193	68.9
	Others	7	2.5
What aspects of your dental treatment were influenced by social media?	Choice of treatment	189	67.5
	Choice of dentist	70	25.0
	Others	21	7.5
Do you believe the results shown on social media are realistic and easily achievable?	Yes	24	8.6
	No	174	62.1
	Not sure	74	26.4
	Others	8	2.9
Do you trust your dentist more than online information for treatment planning?	Yes	207	73.9
	No	38	13.6
	Not sure	31	11.1
	Others	4	1.4
Would you be disappointed if your treatment results don’t match with what you saw online?	Yes	68	24.3
	No	136	48.6
	Maybe	75	26.8
	Others	1	0.4
Have you undergone any restorative or aesthetic dental treatments before, after seeing the same on social media?	Yes	41	14.6
	No	215	76.8
	Others	24	8.6
What type of aesthetic and restorative treatment are you currently interested in?	Silver Amalgam	84	30.0
	Tooth coloured composite filling	126	45.0
	Ceramic veneers	28	10.0
	Bleaching	28	10.0
	Crowns	14	5.0
What do you think is the biggest impact social media has on dental care?	Awareness	94	33.6
	Education	62	22.1
	Real expectations	25	8.9
	Confusions	64	22.9
	Fear	11	3.9
	Unaffordability	23	8.2
	Others	1	0.4
What is your top priority when choosing a dental treatment?	Cost	137	48.9
	Appearance	40	14.3
	Durability	42	15.0
	Time taken	17	6.1
	Dentist recommendation	44	15.7
How do you feel about dental treatments after watching it on social media?	Fearful	34	12.1
	Anxious	71	25.4
	Inspired	84	30.0
	Confused	84	30.0
	Others	7	2.5
For getting a restoration, how much are you willing to spend?	Hundreds	177	63.2
	Thousands	79	28.2
	Lakhs	17	6.1
	Others	7	2.5
Have you seen any social media content claiming that restorative treatments have side effects on your overall health?	No	56	20.0
	Yes	200	71.4
	Others	24	8.6
What all have you seen on social media about silver amalgam fillings?	Good & durable	118	42.1
	Toxic	43	15.4
	Allergic	52	18.6
	Interfere with MRI	29	10.4
	Avoid in pregnancy	38	13.6

The distribution of participants based on gender revealed a higher participation of female subjects (65%, n=182), and the majority of participants (80.0%, n=224) belonged to urban areas. Nearly half of the participants (49.3%, n=138) had completed secondary education, making it the most common educational category, and the majority of the participants possessed at least secondary-level education. Students formed the largest occupational group in the study population, comprising (52.5%, n=147) of participants (Table 1). The distribution of responses regarding exposure to dental-related content on social media revealed that nearly half of the participants (49.6%, n=139) reported seeing such content rarely, indicating that although social media exposure to dental content was common, regular daily exposure was comparatively less among the study population (Table 2).

Instagram emerged as the most commonly used social media platform among the participants, accounting for 42.1% (n=118) of responses. YouTube, Facebook, and WhatsApp were also frequently used platforms. These findings suggest that visually oriented platforms such as Instagram and YouTube play a major role in the dissemination of dental-related content. More than half of the participants (58.2%, n=163) reported that they did not search social media for dental treatment-related information, which indicates that although social media is widely used, it may not necessarily be the primary source of dental treatment information for many individuals. The majority of participants (63.2%, n=177) stated that they did not follow dentists or dental influencers on social media, which indicates that direct engagement with professional dental content creators remains relatively limited among the study population. Regarding the extent of influence of social media on restorative and esthetic dental treatment decisions, strong influence was reported by only 4.6% (n=13) of participants, indicating that social media exerts a mild to moderate impact on treatment-related interest among participants (Table 2).

A majority of participants (68.9%, n=193) reported that they had not tried any dental products after seeing them on social media. Among those who had tried products, whitening strips or pastes were the most common (12.9%, n=36), which demonstrates that although social media exposure exists, actual adoption of promoted dental products remains limited. Social media predominantly influenced the choice of dental treatment, as reported by 67.5% (n=189) of participants. A majority of participants (62.1%, n=174) believed that treatment results shown on social media were not realistic or easily achievable, which suggests that most participants maintained a cautious or sceptical attitude toward idealized treatment outcomes presented online. Most participants (73.9%, n=207) trusted their dentist more than online information for treatment planning. These findings highlight the continued importance of professional dental advice despite widespread exposure to social media content. But unrealistic expectations influenced by social media may affect a subset (24.3%, n=68) of patients. The majority of participants (76.8%, n=215) had not undergone restorative or esthetic dental treatment after seeing similar procedures on social media, suggesting that although social media may generate awareness, it does not always translate into treatment-seeking behaviour (Table 2).

Tooth coloured composite fillings were the most preferred treatment option among participants (45.0%, n=126), followed by silver amalgam restorations (30.0%, n=84). Interest in ceramic veneers and bleaching procedures was equal at 10.0% (n=28) each, while crowns accounted for 5.0% (n=14). These findings reflect a greater preference for conservative and esthetically acceptable restorative options. Awareness creation was identified as the most significant impact of social media on dental care by 33.6% (n=94) of participants (Table 2). Smaller proportions associated social media with unrealistic expectations, fear, or unaffordability. This indicates that social media is perceived both as an educational tool and as a potential source of misinformation or confusion.

Cost was identified as the primary consideration while choosing dental treatment by 48.9% (n=137) of participants, indicating that financial considerations remain a major determinant in treatment decision-making. Conservative spending preferences toward restorative dental care among the participants were noted. The most commonly reported emotional responses after viewing dental treatment-related content on social media were inspiration and confusion, which indicates that social media content can evoke both positive motivation and uncertainty among viewers. Most participants (71.4%, n=200) reported having seen social media content claiming that restorative dental treatments may have adverse effects on overall health. These findings indicate widespread circulation of potentially alarming or misleading dental health information on social media platforms. The most common perception regarding silver amalgam fillings was that they are good and durable, as reported by 42.1% (n=118) of participants. However, a considerable number believed that amalgam restorations may cause allergies (18.6%, n=52), toxicity (15.4%, n=43), interfere with MRI scans (10.4%, n=29), or should be avoided during pregnancy (13.6%, n=38) (Table 2). These findings demonstrate the presence of varied and potentially conflicting information regarding amalgam restorations on social media platforms.

A statistically significant association was observed between educational status and frequency of exposure to dental-related content on social media. Participants with secondary and graduate/professional education reported comparatively higher exposure to dental-related social media content than other educational groups (Table 3). A statistically significant association was identified between educational status and the most commonly used social media platform among the participants. Instagram usage was more prevalent among participants with graduate and professional educational qualifications, whereas WhatsApp and Facebook usage were comparatively higher among participants with lower educational status. A highly statistically significant association was observed between occupation and the most commonly used social media platform. Instagram usage was predominantly observed among students, whereas YouTube and WhatsApp usage were comparatively more common among office workers and housewives.

**Table 3:** Association of sociodemographic variables with social media usage patterns related to dental content *Statistically significant at p < 0.05, Fisher’s Exact Test.

Variable	Outcome Assessed	χ² Value	p-value
Educational Status	Frequency of exposure to dental-related content on social media	34.855	0.021*
Educational Status	Most commonly used social media platform	42.617	0.002*
Occupation	Frequency of exposure to dental-related content on social media	45.303	0.036*
Occupation	Most commonly used social media platform	63.973	<0.001*
Socioeconomic Status	Searching social media for dental treatment information	31.551	0.007*
Age group	Most commonly used social media platform	46.371	<0.001*

No statistically significant association was observed between educational status and searching social media for dental treatment-related information. No statistically significant association was observed between occupation and searching social media for dental treatment-related information, although students constituted the largest proportion searching for dental information online. No statistically significant association was observed between socioeconomic status and frequency of exposure used to dental-related content or commonly used social media platform. No statistically significant association was identified between age group and frequency of exposure to dental-related content on social media or between age group and searching social media for dental treatment-related information. A statistically significant association was found between occupation and frequency of exposure to dental-related content on social media. Students and professionals demonstrated comparatively higher exposure to dental content than other occupational groups. A statistically significant association was observed between socioeconomic status and searching social media for dental treatment-related information. A highly statistically significant association was observed between age group and the most commonly used social media platform (Table 3).

## Discussion

This study evaluates the role of social media in influencing patients’ expectations from esthetic restorative treatments and the gap between real and digital dentistry, taking into account the demographic patterns and trust dynamics. Dental esthetics play an important role in the perception of a visually pleasing face, with the younger population showing a greater awareness of dental esthetics [5]. Recently, there has been an increase in the demand for dental treatment for esthetic purposes, mainly influenced by the appearance of public figures and social media. Abbasi et al. observed that many dentists believe that patients visiting dental clinics are seeking esthetic dental treatment because of their popularity on social media [6]. However, it is also important to consider the influence of social media from the patients’ perspective. Therefore, this study explored the influence that social media has had on the demand for esthetic treatments. A comprehensive literature review was conducted to identify previously published questionnaires related to this study [3,6]. Relevant items from existing validated questionnaires were reviewed for their content, relevance, and applicability to the objectives of the present study. Based on this review, a study-specific questionnaire was developed by adapting and integrating relevant items and incorporating additional questions considered necessary to address the specific objectives and context of the study.

The social media's role in dentistry is heavily dictated by demographic variables. The over-representation of female responders could also be associated with female curiosity about appearance-related services, as reported in the past gender-specific esthetic literature. This is consistent with global trends, where we find ourselves becoming increasingly reliant on social platforms for health information, and patients are using them as a means of acquiring health information [7]. A statistically significant association was found between educational status and the frequency of exposure to dental content (p = 0.021), with secondary and graduate-level participants reporting the maximum engagement. Occupation also seems to significantly dictate the platform choice, with Instagram being the dominant medium for students and highly educated professionals, whereas YouTube, WhatsApp, and Facebook are more prevalent among participants with lower educational backgrounds or different occupational statuses, such as office workers and housewives. This alignment of visually oriented platforms like Instagram (42.1%) and YouTube (19.3%) with younger, educated demographics suggests that these platforms are the primary influencers of esthetic trends. So, these media can be used to access specific subsets of the population for communication of health-related facts by certified professionals.

About 73.9% of patients still trust their dentist more than online information for treatment planning. Dentists should leverage this high level of trust to steer patients away from trends and move towards evidence-based treatment modalities. These findings underscore the importance of online reputation management and indicate that social media-based reviews and patient feedback indeed can have a major influence on clinical footfall, consistent with earlier digital marketing research in health care [8]. This is consistent with recent literature that indicates influencer fatigue has created a patient base that demands authenticity over ads [9]. Interestingly, the source of digital trust has shifted from celebrity endorsements to peer authenticity. Dentists must act as mediators to bridge the gap between digital content and patient expectations. Dental education and health promotion should be done by certified dentists or health professionals by actively using social media platforms to provide accurate and scientific information. Lack of a peer review process on these platforms can lead to the dissemination of inaccurate and potentially misleading health information [10]. Influencers and celebrity figures must strictly adhere to Advertising Standards Council of India (ASCI) guidelines and distinguish between personal wellness views and professional medical advice [11]. They are bound to provide a clear disclaimer clarifying that their content should not be seen as a substitute for professional medical advice. Influencers must clearly state their professional qualifications in any content related to health, fitness, or nutrition. These influencers must be aware that non-compliance and sharing misleading, false, or harmful health information can lead to penalties under the Consumer Protection Act 2019.

Previous studies showed that 41.4% had visited their dental clinic’s social media page and 36% had searched for their dentist on social media [12]. 24.3% of patients would feel disappointed if their clinical results did not match digital representations. This disappointment is due to the quick-fix, simplified nature of social media videos, which may comfort anxious patients but often dilute the complexity of actual procedures. Proactive expectation management should be done in the initial dental visit by correcting unrealistic expectations and counselling patients against unnecessary cosmetic procedures [13]. Although social media serves as a platform for patient awareness and information dissemination, its influence on actual treatment decisions appears to be moderate. Treatment cost remains the primary determinant for 48.9% of patients, suggesting that financial constraints often override the esthetic desires fuelled by digital media. Dentists must act as mediators by using social media platforms for ethical patient education and directly addressing common myths regarding material safety. American Dental Association Council on Scientific Affairs states that studies continue to support that dental amalgam is a safe restorative option for both children and adults. When responding to safety concerns, it is important to make the distinction between known and hypothetical risks [14].

Social media works at once as an instrument of patient education and as a driver of distorted esthetic expectations, and the clinician stands at the point where those two forces meet. The clinical and ethical response rests with the dentist, who must manage expectations, secure genuinely informed consent, screen for body image disturbance, and uphold honest, professional communication [15]. It is important for the use of social media by dental professionals to fit within the established social contract between the profession and society, and failure to observe the terms of this will cause damage to the patient-professional relationship [16]. A cross-sectional study surveyed 515 adults in Saudi Arabia to evaluate how exposure to esthetic dentistry content on social media affects self-esteem and smile satisfaction. They found that Snapchat and Instagram are the primary platforms for viewing dental content, with personal recommendations and promotional discounts acting as the main motivators for seeking treatment. Sociodemographic factors like age, gender, occupation, and monthly income were significantly associated with participants' overall smile satisfaction and their willingness to share personal photos online [17]. A similar study conducted by A Vinita Mary et al. on the impact of social media on dental treatment needs found that 50.3% occasionally encountered dental information on social media, while 28.9% never saw such content. A significant majority didn’t make their dental treatment decisions on recommendations from social media, while around 36.9% were neutral or showed no interest in this matter [18].

Social media can be used for professional networking and positive self-promotion. Healthcare professionals should utilize social networking to educate and communicate with the community [19]. A cross-sectional study on the psychological influence of social media on young people’s self-perception of smile and the demand for cosmetic dental procedures found that young people who reported higher engagement with appearance-related content on digital platforms also tended to report greater awareness of smile esthetics and interest in esthetic dental treatments. These findings highlight the potential influence of digital environments on self-perception during adolescence, a developmental stage characterized by heightened sensitivity to peer opinions and social comparison [20].

## Conclusions

Social media can be used effectively by certified professionals to spread awareness about dental health and treatment options. Visual platforms like Instagram and Facebook can be used to disseminate information to different subsets of the population, but the influence on actual decision-making appears to be moderate. Social media is a double-edged tool as it can act as an educational resource that increases patient awareness and reduces anxiety through informative videos, yet it simultaneously propagates unrealistic esthetic standards and health-related misinformation. There should be strict monitoring against the circulation of misleading health information and measures to ensure adherence to ASCI guidelines. Ethical and accurate information by qualified professionals can bridge the gap between digital portrayals and realistic clinical outcomes, thereby improving patient understanding, trust, and treatment satisfaction. Dentists must act as mediators to bridge the gap between digital content and patient expectations.
